# Multidimensional context of technical nursing education: triangulation of meanings attributed by teachers^*^

**DOI:** 10.1590/1980-220X-REEUSP-2021-0513en

**Published:** 2022-07-08

**Authors:** Bruna Sabrina de Almeida Sousa, Benevina Maria Vilar Teixeira Nunes, Fernando Rocha Porto, Inez Sampaio Nery, Maria Eliete Batista Moura, Herica Emilia Félix de Carvalho

**Affiliations:** 1 Universidade Federal do Piauí, Programa de Pós-graduação em Enfermagem, Teresina, PI, Brazil.; 2 Universidade Federal do Estado do Rio de Janeiro, Programa de Pós-Graduação em Enfermagem, Rio de Janeiro, RJ, Brazil.; 3 Universidade de São Paulo, Escola de Enfermagem de Ribeirão Preto, Programa de Pós-Graduação em Enfermagem Fundamental, Ribeirão Preto, SP, Brazil.

**Keywords:** Education, Nursing, Faculty, Nursing, Licensed Practical Nurses, Social Support, Students, Nursing, Educación en Enfermería, Docentes de Enfermería, Enfermeros no Diplomados, Apoyo Social, Estudiantes de Enfermería, Educação em Enfermagem, Docentes de Enfermagem, Técnicos de Enfermagem, Apoio Social, Estudantes de Enfermagem

## Abstract

**Objective:**

To unveil and discuss the meanings attributed by teachers to the teacher-student relationship in the multidimensional context of technical nursing education.

**Method:**

Qualitative research, supported by the representational theory of meaning, carried out with nine nurses who teach the technical nursing course at a technical school linked to a federal university in the northeastern region of Brazil.

**Results:**

The following meanings were revealed: student profile; lack of preparation and financial resources; reduction in performance and even course interruption; students’ disinterest and resistance; academic support and qualified structure; opportunity for grants; teachers’ dedication and holistic view; respect, ethics, and interactivity; concern with student learning and performance, and heterogeneous group.

**Conclusion:**

Social and financial difficulties and students’ disinterest are the limitations that most hinder teaching effectiveness. On the other hand, the teachers’ holistic view, dedication, support, and the availability of resources and academic support are the main multidimensional characteristics of living in this scenario.

## RESUMO

**Objetivo:**

Desvelar e discutir os significados atribuídos por docentes à relação entre professor e aluno no contexto multidimensional do ensino técnico de enfermagem.

**Método:**

Pesquisa qualitativa, apoiada na teoria representacional do significado, realizada com nove enfermeiros docentes do curso técnico em enfermagem de um colégio técnico vinculado a uma universidade federal da região nordeste do Brasil.

**Resultados:**

Foram desvelados os significados: perfil dos alunos; falta de preparo e de recursos financeiros; queda do rendimento e até interrupção da formação; desinteresse e resistência dos alunos; suporte acadêmico e estrutura qualificada; oportunidade de bolsas de auxílio; dedicação e olhar holístico dos docentes; respeito, ética e interatividade; preocupação com o aprendizado e desempenho do estudante, e grupo heterogêneo.

**Conclusão:**

Dificuldades sociais, financeiras e o desinteresse estudantil são as limitações que mais dificultam a efetividade do ensino. Por outro lado, o olhar holístico, a dedicação, o apoio dos docentes e a disponibilidade de recursos e suporte acadêmico são as principais características multidimensionais da vivência nesse cenário.

## RESUMEN

**Objetivo:**

Desvelar y discutir los significados atribuidos por docentes sobre la relación entre profesor y alumno en el contexto multidimensional de la enseñanza técnica en enfermería.

**Método:**

Investigación cualitativa, apoyada en la teoría representacional del significado, realizada con nueve enfermeros docentes del curso técnico en enfermería de un colegio técnico vinculado a una universidad federal de la región noreste de Brasil.

**Resultados:**

Fueron desvelados los significados: perfil de los alumnos; falta de preparación y de recursos financieros; baja del rendimiento e incluso interrupción de la formación; desinterés y resistencia de los alumnos; soporte académico y estructura calificada, oportunidad de becas de auxilio; dedicación y mirada holística de los docentes, respeto, ética e interacción, preocupación por el aprendizaje y desempeño del estudiante y grupo heterogéneo.

**Conclusión:**

Dificultades sociales, financieras y el desinterés del alumnado son las limitaciones que más dificultan la efectividad de la enseñanza. Sin embargo, la mirada holística, la dedicación, el apoyo de los docentes y la disponibilidad de recursos y soporte académico, son las principales características multidimensionales de la vivencia en ese escenario.

## INTRODUCTION

The teaching-learning process is permeated by changes occuring according to the population’s demands. Thus, student and professional training has a multidimensional amplitude, since knowledge influences the individuals’ educational, social, and cultural development. Teaching guiding health care also involves such aspects; however, there is currently a challenge to address the biopsychosocial dimensions in the student’s environment^(1)^.

Teaching at a technical level has changed in recent years, and traditional mechanized training has been replaced by methods with an emphasis on skills, quality, and criticality. However, this trend has not yet fully materialized in reality because, over time, due attention has not been paid to the pedagogical training of nurses working in the training of new professionals. In fact, teachers themselves have not demonstrated any concern regarding their weakened performance^(2,3)^.

Teaching performance fragility has a negative impact on pedagogical practice. Constantly, discouragement, dissatisfaction, personal demotivation, devaluation, daily physical exhaustion, disenchantment with the process of construction of knowledge and with teaching itself can be observed in the nurse teacher daily routine. This leads to suffering, as the frustrated individual sees his personal and professional trajectory shaken, which interferes with didactics and his/her ability to improve in the face of external context influences^(3,4)^.

Teachers are important agents in society’s processes of change. This means that they need investments from the appropriate institutions, in training and professional development, for quality performance and to master innovative didactic-pedagogical methodologies, capable of offering positive results in the students’ development. Therefore, teachers are mediators among students, education, and the field of work. Therefore, its role shall meet the social demands^(5,6)^.

Thus, the teacher needs to understand the broad notion of training that encompasses multiple dimensions, such as the educator’s preparation, the student’s profile, the teaching methodology, the didactic contents, the teaching strategies, the evaluation of learning, the cultural aspects, and the influence of interpersonal relationships in secondary education in nursing. Everyday life in the field of education is permeated by social experiences, and teachers can enjoy daily contact to get to know and understand students and their demands, seeking to improve teaching practice^(7)^.

Teachers of the technical nursing course are relevant for teaching as well as for health care, as they are responsible for training those who serve the population and form the basis of care. Therefore, the teacher’s role is complex and related to the commitment to share knowledge with future professionals, helping them in their educational and social emancipation. This explains the representativeness of teacher-student interaction, which prompts the following question: what are the meanings of the teacher-student relationship in the multidimensional context of technical nursing education?

We emphasize the scarcity of studies that, like this one, approach the technical nursing education under the educational, social and reflexive dimensions, presenting the interpersonal relationship between teacher and student, based on meanings. This multidimensional perspective is little explored and deserves to be highlighted, as it reveals various aspects that interfere in teaching. Current studies approach the technical nursing course only with regard to training and pedagogical practice^(2,3,6–9)^. Therefore, the objective of this study is to unveil and discuss the meanings attributed by teachers to the teacher-student relationship in the multidimensional context of technical nursing education.

## METHOD

### Design of Study

This is a qualitative research, supported by Ogden and Richards’ Representational Theory of Meaning (RTM).

### Population

The participants were nine teaching nurses of the nursing technical course belonging to a technical school of reference that is linked to the Federal University, located in one of the capitals of the northeastern region of Brazil. It is a faculty consisting mostly of women (77.7%), with more than 10 years of training and experience in nursing (100%), with four doctors, two post-docs, two masters, and one specialist, all with experience in teaching, research, and extension. The invitations to participate took place in person, in the teachers’ work environment.

### Selection Criteria

Nurses who had been teaching the aforementioned technical course for at least one year were included in the study. It should be noted that all teachers met the inclusion criteria. As a result, exclusion criteria were not applicable.

### Data Collection

Data production took place from January to April 2019, through interviews conducted privately, by a research nurse with experience in nursing education topics, who had no previous relationship with the interviewees. A semi-structured script was used, with an open questioning, in which the participants could freely discuss teaching in technical nursing education, and the audio contents were recorded on an electronic device.

Then, the recorded reports were fully transcribed and, to preserve anonymity, it was decided that the nurses’ names would be replaced with the expression “Teacher” followed by the number corresponding to the order of participation during the interviews. In this study, the criteria established in the *COnsolidated criteria for REporting Qualitative research* – COREQ, a support protocol for studies with qualitative methods^(10)^ were followed.

### Theoretical and Methodological Basis

**Figure 1.**
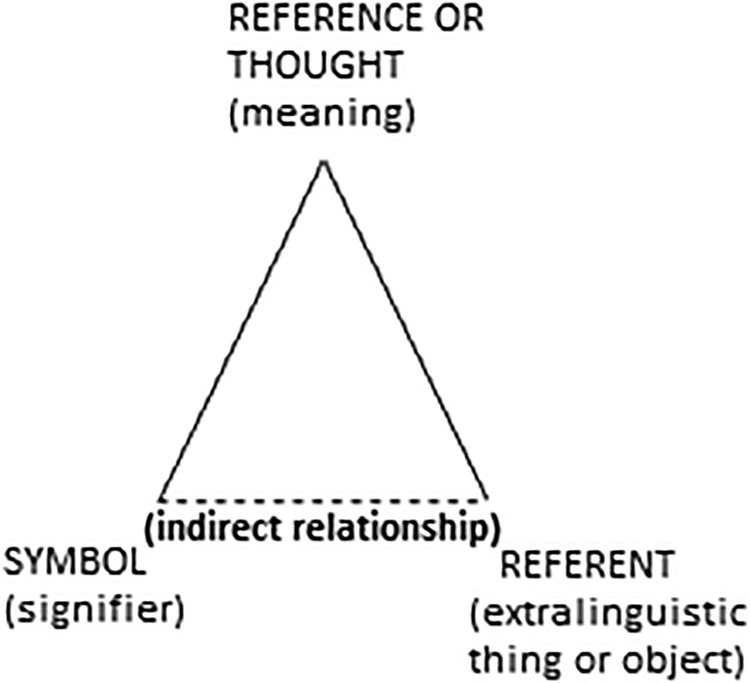
Meaning Triangle^(12)^ (Source: Littlejohn, 1982).

The reports were analyzed and, based on them, linguistic units organized according to the themes addressed by the teachers during the interviews were identified. Ogden and Richards’ Representational Theory of Meaning was used, which brings the interconnection of three elements in the development of the sense of meaning, to compose the “Semantic Triangle” shown in Figure 1 ^(12)^ with: the symbol (what the word means), the referent (the meaning of this linguistic thing/object) and the reference or thought (what it means to the person)^(11,12)^.

The relationship between the symbol and the referent is associated with a person’s thought. The interconnection of these aspects results in the creation of a meaning, in which it can acquire numerous concepts in communication; however, from the RTM, the one with the greatest representational meaning is adopted^(11)^.

After reading the teachers’ reports, the referents and symbols of the written stretches were extracted, according to the themes found; these evidenced a thought. When analyzing the association of the three elements together, the meaning of what was said by the research participants became clear.

### Ethical Aspects

The research complied with the ethical principles of Resolution nº 466/2012 of the National Health Council – CNS^(13)^ and the precepts of Resolution No. 510/2016 of the CNS, on standards applicable to research in the human and social sciences^(14)^. The research was approved by the Research Ethics Committee on September 28, 2018, with Opinion No. 2.927.504.

## RESULTS

Following the methodological proposal, we created a demonstrative chart to highlight the Semantic Triangle that emerged from the nine research participants’ speeches.

**Chart 1.**
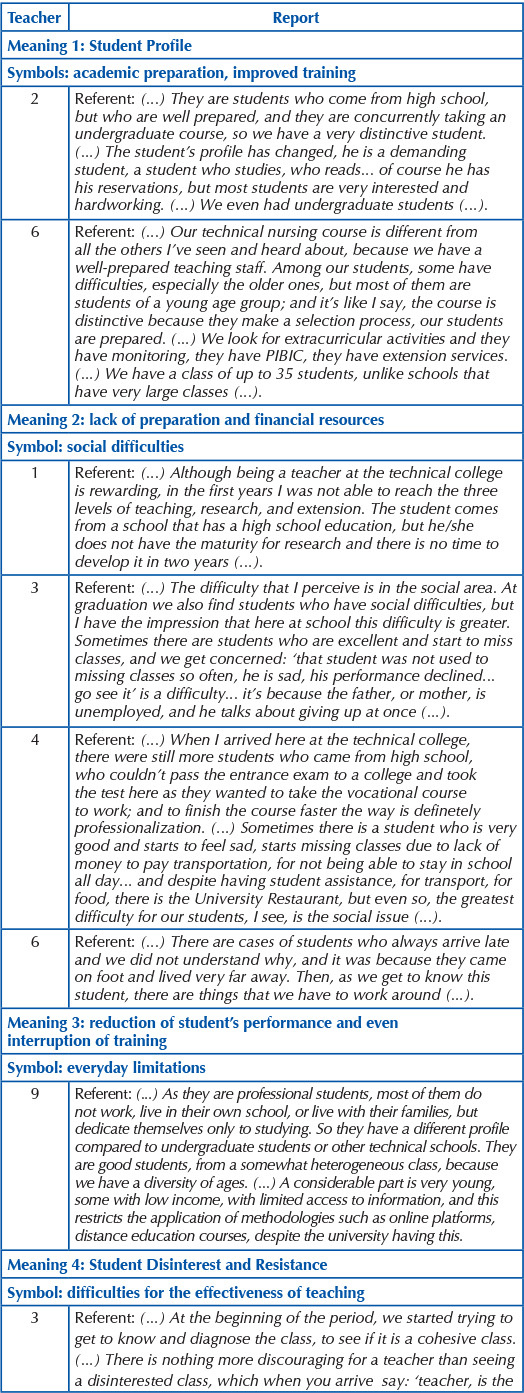

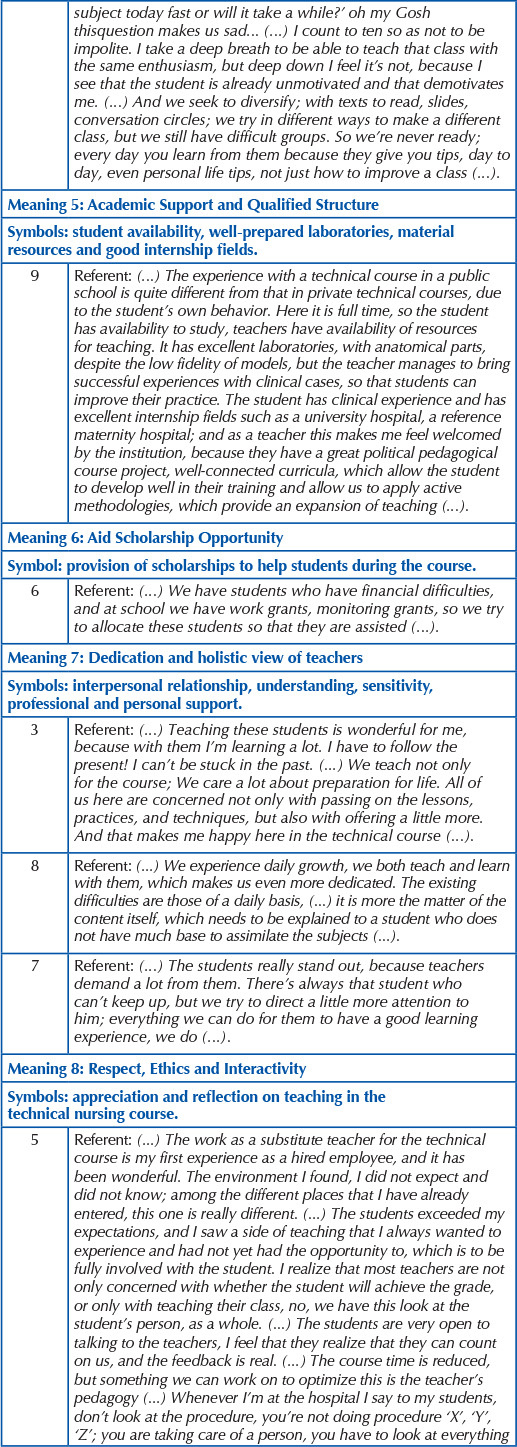

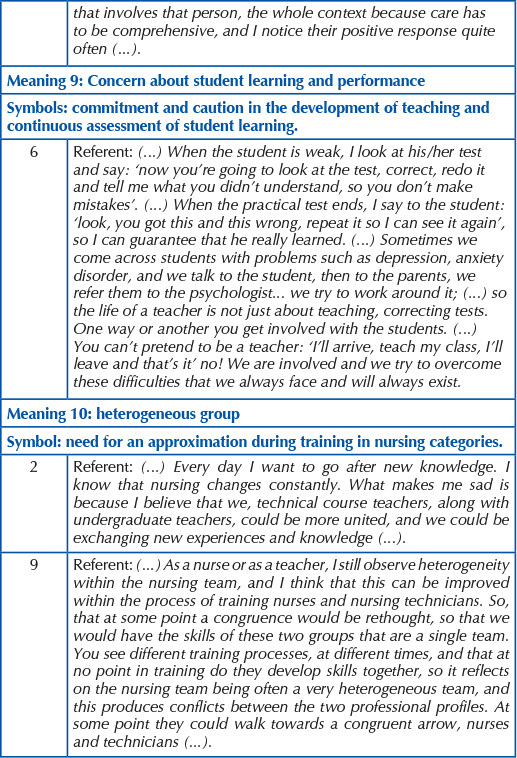
Symbol and referent in the construction of thought: meanings – Teresina, PI, Brazil, 2019.

Chart 1 presents, based on the referents and symbols, ten revealed meanings that reflect the thoughts of the interviewees according to their reports. The following discussion was developed from this perspective and deals with information that sequentially highlights each of the ten meanings.

## DISCUSSION

Based on a contemporary conception, it is known that the educators’ job begins the moment they assume that their mission goes beyond the professional activity, since the focus of the act of teaching is not the results and final sums, but the learning, the acquired experiences and the interpersonal relationships that are built and that facilitate the teacher, student, and teaching triad. Thus, education constitutes one of the pillars of human development, by virtue of transcending the parameters of teaching and strikingly incorporating itself into the social scenario.

A society in constant transformation has different problems in the financial, social, political, or ideological sphere; however, knowledge opens new horizons for a fairer social environment with more opportunities. Teachers expressed that, in general, students have a profile with good academic performance (meaning 1). However, this fact reveals a discrepancy when we are faced with a frequent problem that impacts the course: social difficulties. From the teachers’ view, the social issue represented by the lack of resources is one of the biggest barriers to the teaching-learning process (meaning 2).

Parents’ unemployment, financial limitations for public transport, and restricted access to information were some of the mentioned symbols that usually cause sadness and withdrawal. Furthermore, the resistance of some students, the immaturity for research and extension, and the limited time of the course were other aspects mentioned. The reports reveal problems that students deal with on a daily basis. Many students leave small towns to study; however, they go through an adaptation process and carry deficiencies with them that can result in a reduction of performance and even course interruption^(15)^.

Limited access to the internet is one of the factors that can hinder the course smooth running (meaning3). Similar to what was exposed in the results, the participants of a study pointed out that the absence or poor quality of the internet is a factor that makes it difficult to participate in distance education or blended courses^(16)^. Greater access to the internet and digital platforms is something that can be extremely beneficial for teaching and learning if the student uses interactive tools to search for knowledge^(8)^. In a survey of nursing students, 91.4% of them used materials from the internet in their studies^(17)^.

Another aspect revealed is the students’ lack of interest and resistance regarding teaching, which leads to teachers’ discouragement (meaning 4). This datum is analogous to that found in a survey carried out with nurses who teach technical courses, which pointed out that the teacher feels disrespected and devalued in the face of students’ lack of commitment to training^(4)^. To encourage students in class, teachers seek teaching strategies to streamline learning, such as the use of active methodologies that facilitate learning and interactivity. Allied to this, educators need to be proactive, creative, and innovative in pedagogical practice, aiming at horizontal communication and teaching quality^(18,19)^.

Student availability, well-prepared laboratories, quality material resources and internship fields were aspects highlighted by “teacher 9”, who mentions that they bring confidence to the teacher’s practice, something relevant to the teaching quality (meaning 5). The equipment available for the classes allows the use of methodologies that develop theoretical and clinical skills.

In a study carried out in South Africa, regarding the implementation of problem-based learning, the authors highlighted that this methodology can bring together the aspects of training and nursing care, benefiting the workforce^(20)^. Innovative and interactive methodologies stimulate individual and collective reasoning and critical thinking, associating theory with professional practice. Considering that quality teaching comes from well-structured proposals, “Teacher 9” also informed that the technical course has a well-designed pedagogical project, which contributes to the teaching and learning process being developed with planning and efficiency.

On the other hand, in a study carried out with 15 teachers, it was found that 46.7% do not know the pedagogical project of the course in which they work, which means that the teaching practice is not in line with the guidelines proposed by the institution for the educational process^(7)^. It is essential that the educator knows the field pedagogical policy, as well as the student’s profile, to understand the perspectives of the profession and offer qualified and updated training^(21)^.

At school, students also have opportunities to receive a tutoring grant, a work grant, residency at the school and institutional food service at the university restaurant, as well as the teachers’ support and assistance. These assistance opportunities offered by the institution mean support for students (meaning 6).

In addition, aiming at settling the obstacles encountered in the course’s day-to-day, the teachers emphasize the importance of supporting the student and maintaining a holistic view (meaning 7). The teachers reveal the attention and helpfulness they direct to their students, reinforcing the concept that care is born from human relationships and is characterized by attention to the other and by interaction, which awakens empathy and the construction of affective bonds^(22)^.

Empathy, understanding, patience and respect are characteristics understood as socio-emotional skills necessary for teachers of a technical nursing course, considering the relevance of these aspects^(23)^. In this regard, the bond between teachers and students shall be mediated by embracement, appreciation of knowledge, humanization and dialogue, as these aspects are the key pieces for the construction of successful interpersonal relationships^(9)^.

The participants assume an embracing posture as they turn their eyes to the students’ social difficulties, and try to overcome them in the best way. Through the interviews, it was noted that teachers value involvement with students and seek, daily, to listen and support them in their experiences, and not only in the professional aspect (meaning 8). An international study brings in its data that the teaching-learning process becomes easier in an environment in which students feel comfortable to express themselves and manifest their aspirations and thoughts, as the development of relationships must be based on respect, ethics, and interactivity^(24)^.

Based on the reports, the teachers of technical nursing education, besides keeping a holistic view of their students, strive to transmit this perspective to them, so that they later become professionals capable of attending to the patients’ singularities. The student has to develop a reflective look at the nature of care, so that they can understand the practical reality of the nursing field^(25)^.

The concern with student learning and performance also demonstrates the ethical and committed attitude from teachers, who seek to prove the success of their teaching (meaning 9). A research that addresses Technical Schools of the Brazilian Public Health System (*ETSUS*) in the Northeast states that these institutions have an evaluation method and chances of recovery, offering an opportunity for the student to improve deficits and strengthen learning^(26)^.

An Iranian study on clinical nursing education found that after students performed practical procedures, clinical educators did not provide feedback to them about their performance, making students feel insecure about their abilities^(27)^. In view of this, it is pertinent that the teacher knows how to provide the student’s intellectual and practical independence, through training that allows the wide development of skills, so that the student has autonomy, attitude, and flexibility to act in different situations that may arise in their experience^(28)^.

In technical education, teachers shall daily encourage the development of their students in a comprehensive way, understanding them as social subjects who must be prepared not only as students, but as citizens and future professionals^(9)^. The human being is inseparable from its inherent characteristics, so these must be involved in the development process, not suppressed.

After reflecting on the pedagogical experiences, the reports showed that the nursing profession corresponds to a heterogeneous group, composed of workers from different categories, but who work in the same team (meaning 10). The stratification of work is the result of a historical conception in which care is arranged between supervision and assistance, giving rise to different training processes over time^(25,29)^. Therefore, the teachers pointed out that the training of nursing workers requires an approximation, as professional practice is carried out in a team, and this requires symmetry within the groups.

## CONCLUSION

From the reports obtained, ten meanings were revealed about the teacher-student interpersonal relationship in the technical nursing course, which allowed us to understand that social and financial difficulties and the student’s lack of interest are the limitations that most hinder teaching effectiveness. On the other hand, the holistic view, dedication, support from teachers and the availability of resources and academic support are the main multidimensional characteristics of living in this scenario. Through these attributes, teachers daily seek to circumvent the obstacles encountered and offer quality education, aiming at the development of knowledge and citizenship.

As a limitation of the study, we highlight the fact that the technical school has an excellent structure for teachers, and that entry into the work field occurs through a public test, and represents financial stability. These positive aspects may have influenced the teachers’ critical view in relation to existing difficulties, as the teacher may have felt uncomfortable to report them in full.

In short, this study contributes to the scientific community by exposing the psychosocial relevance of the mission of educating, considering that teachers of technical nursing education are important agents in the edification of ethical citizens and in the training of new professionals who will be responsible for the population’s health care. Further studies in the area are recommended to broaden the discussion on technical nursing training, and so that it is possible to understand even more the teachers’ pedagogical experiences and the students’ needs.

## ASSOCIATE EDITOR

Vilanice Alves de Araújo Püschel
